# Comparison of nutritional risk screening with NRS2002 and the GLIM diagnostic criteria for malnutrition in hospitalized patients

**DOI:** 10.1038/s41598-022-23878-3

**Published:** 2022-11-17

**Authors:** Marte A. Trollebø, Eli Skeie, Ingrid Revheim, Helene Stangeland, Mari-Anne H. Erstein, Martin K. Grønning, Randi J. Tangvik, Mette H. Morken, Ottar Nygård, Tomas M. L. Eagan, Hanne Rosendahl-Riise, Jutta Dierkes

**Affiliations:** 1 Center for Nutrition, University of Bergen, Bergen, Norway; 2 Mohn Nutrition Research Laboratory, University of Bergen, Bergen, Norway; 3 Department of Clinical Medicine, University of Bergen, Bergen, Norway; 4 Department of Clinical Science, University of Bergen, Bergen, Norway; 5 Department of Heart Disease, Haukeland University Hospital, Bergen, Norway; 6 Department of Thoracic Medicine, Haukeland University Hospital, Bergen, Norway; 7 Department of Clinical Nutrition, Haukeland University Hospital, Bergen, Norway; 8 Department of Research and Development, Haukeland University Hospital, Bergen, Norway; 9 Department of Medical Biochemistry and Pharmacology, Haukeland University Hospital, Bergen, Norway

**Keywords:** Nutrition, Diagnosis

## Abstract

Nutritional risk screening, to identify patients at risk of malnutrition, is the first step in the prevention and treatment of malnutrition in hospitalized patients, and should be followed by a thorough nutritional assessment resulting in a diagnosis of malnutrition and subsequent treatment. In 2019, a consensus on criteria has been suggested for the diagnosis of malnutrition by the Global Leadership Initiative for Malnutrition (GLIM). This study investigates the diagnosis of malnutrition in hospitalized patients using nutritional risk screening and the diagnostic assessment suggested by GLIM. Hospitalized patients (excluding cancer, intensive care, and transmissible infections) who underwent nutritional risk screening (by NRS2002) were included. Nutritional risk screening was followed by anthropometric measurements including measurement of muscle mass, assessment of dietary intake and measurement of serum C-reactive protein (CRP) for inflammation in all patients. Malnutrition was diagnosed according to the GLIM-criteria. In total, 328 patients (median age 71 years, 47% women, median length of stay 7 days) were included. Nutritional risk screening identified 143 patients as at risk of malnutrition, while GLIM criteria led to a diagnosis of malnutrition in 114 patients. Of these 114 patients, 77 were also identified as at risk of malnutrition by NRS2002, while 37 patients were not identified by NRS2002. Malnutrition was evident in fewer patients than at risk of malnutrition, as expected. However, a number of patients were malnourished who were not identified by the screening procedure. More studies should investigate the importance of inflammation and reduced muscle mass, which is the main difference between nutritional risk screening and GLIM diagnostic assessment.

## Introduction

In European hospitals, continuous effort towards the identification and the treatment of disease-related malnutrition (DRM) is going on to improve health care efficiency by securing patients’ nutritional status^1^. DRM is an etiology-based type of malnutrition caused by a concomitant disease and can occur with or without inflammation^2^. Malnutrition and the risk of malnutrition are associated with detrimental consequences such as higher rates of complications, longer hospitalization, and increased mortality^3–5^. It is estimated that one-quarter to one-third of hospitalized patients in European hospitals, including Norway, are malnourished or at risk of malnutrition, with an even higher prevalence observed among older patients, and in patients with specific diseases^6–11^.

DRM is well described, and the need to prevent and treat the condition is urgent. However, securing the patients’ nutritional status during hospitalization meets several challenges, including lack of knowledge and routines, unclear responsibilities, and a low number of clinical dieticians accessible^12–14^.

The first important step to identify patients who may be in need of nutritional support is nutritional risk screening. Different tools have been validated for the screening process^15^, and in Norway, usually, NRS2002 is used^16,17^.

In principle, patients at risk of malnutrition identified by nutritional screening should receive a thorough nutritional assessment, a diagnosis of malnutrition, and subsequent treatment. However, few studies compare results of nutritional risk screening with results from the subsequent diagnosis. This may also have been due to a lack of standardized methods, criteria, and cut-off values for diagnosing and classifying malnutrition. In 2019, the Global Leadership Initiative of Malnutrition (GLIM) suggested a two-step approach for diagnosing malnutrition. In the first step, hospitalized patients should be screened for nutritional risk by a validated screening tool. In the second step, the following diagnostic assessment criteria should be used to diagnose malnutrition among the patients who are at risk of malnutrition: presence of at least one phenotypic criterion (weight loss, low body mass index (BMI), and reduced muscle mass) and one etiologic criterion (reduced food intake or assimilation and inflammation). In addition to nutritional screening, assessment of muscle mass and inflammation are taken into account. Suggested methods for assessment of muscle mass include dual-energy absorptiometry, bioelectrical impedance, ultrasound, computed tomography or magnetic resonance imaging, or alternative measures such as mid-arm muscle circumference (MAMC) or calf muscle circumference, and handgrip strength (HGS) as a supportive measure^18^. Both reduced muscle mass and inflammation have been shown to predict outcomes in hospitalized patients^19,20^. Finally, GLIM suggests classifying malnutrition as stage 1 (moderate) or stage 2 (severe) based on the phenotypic criteria^18^. Introducing a diagnostic framework with several proposed methods in the first two steps of the diagnostic process requires studies applying these criteria in clinical practice^21^. Moreover, a previous study on patients with colorectal cancer, suggests that the patient population identified by the GLIM-criteria is also dependent on the screening tool used in the first step of the diagnostic process^22^.

Thus, this study aimed to investigate DRM in a population of patients who have undergone nutritional risk screening with NRS2002 and the process of diagnosing malnutrition following the second step of the GLIM-criteria in a tertiary hospital.

## Subjects and methods

### Study design and study population

The study was conducted at Haukeland University Hospital in Bergen, Norway, in collaboration with the University of Bergen from September 2017 to December 2019. Patients ≥ 18 years who underwent nutritional screening at the ward, with cognitive and linguistic abilities to understand the study purposes and to sign the informed consent form, were eligible for inclusion. Patients undergoing cancer treatment, receiving intensive care, and patients with transmissible infections were excluded. Eligible patients were included after consultation with the nurses at the wards and based on the NRS2002 outcome as performed routinously by the nurses. Recruitment of potential patients was undertaken each workday by study personnel consisting of trained dieticians and a study nurse. There was one study visit per patient, and all data were collected in the patients’ room according to standard operating procedures developed for the study. The study was conducted at the Departments of Thoracic Medicine, Heart Disease, Dermatology/Rheumatology, Gastroenterology, Endocrinology, and the Orthopedic Clinic; the latter four departments are categorized as “other departments” throughout the paper due to the number of patients included. The study procedures included an additional nutritional screening of all patients by trained dieticians followed by anthropometric measurements including measurement of muscle mass, assessment of dietary intake and measurement of serum CRP in all patients.

### Nutritional risk screening using NRS2002

In general, NRS2002 is characterized by two parts: the initial screening consisting of four «Yes»/«No» questions and the main screening^16^. The four initial «Yes»/«No» questions assess low BMI (< 20.5 kg/m^2^), weight loss during the last 3 months, reduced dietary intake during the last week, and if the patient is severely ill. The patient shall proceed to the main screening if at least one question is answered with «Yes». The main screening scores the impairment of nutritional status (0–3 score) and the disease severity (0–3 score). Finally, 1 score is added when the patient is ≥ 70 years, leading to a maximum score of 7. A total score of 3 or higher is regarded as being at risk of malnutrition^16^.

### Study procedures

#### Nutritional risk screening

The nutritional risk screening was performed by trained dieticians and included measurement of weight and height, and a questionnaire addressing reduced food intake the last week and weight loss the last 3 months.

Patients were measured in light hospital clothes without shoes. Bodyweight was measured once using a portable electronic scale (Seca model 877, Hamburg, Germany) to the nearest 0.1 kg. Weight measurements were not possible in 40 patients, and in these cases, self-reported weight was used. Height was measured in centimeters using a portable stadiometer (Seca model 217, Hamburg, Germany) to the nearest 0.1 cm. Patients were asked to stand with arms down, feet together, heels against the backboard, and knees straight, and to look straight ahead (Frankfurt horizontal plane). The mean value of two measurements was used for statistical analysis. In cases where height was not possible to measure, self-reported height was used (n = 62). Bodyweight and height were used to calculate body mass index (BMI) (bodyweight [kg]/height [m]^2^).

### Further assessments

Originally, it was planned to use appendicular muscle mass measured by bioelectrical impedance (BIA) measurements, however, BIA could not be used in 96 patients and therefore mid-arm muscle circumference (MAMC) was used to assess low muscle mass which was calculated from mid-upper arm circumference (MUAC) and skinfold thickness (SFT).

SFT was measured three times in millimeters using a Lange skinfold caliper (Santa Cruz, USA). The mean value was used for further analysis. MUAC was measured twice using an ergonomic measuring tape, Seca model 201 (Hamburg, Germany). Each measurement was noted at the nearest 0.1 cm, and the mean value of the two measurements was used for further analysis. MAMC was calculated using the formula: MUAC- (3.14 × SFT × 0.1). Age and sex-specific cut-offs for MAMC were applied^23^.

HGS was measured by a dynamometer (Jamar Smart, Patterson Medical, Warrenville, IL, USA) in triplicate and is reported as kg. Both average and maximum HGS were recorded, and maximum HGS was used for statistical analysis.

C-reactive protein in plasma (CRP) was used to characterize inflammation. CRP was measured from non-fasting routine morning blood samples taken the day after study inclusion. The samples were analyzed at the central laboratory at the Haukeland University hospital. Information regarding the patients’ diagnosis were found in the discharge letters in the medical records. Length of stay at the ward and all diagnoses were obtained from the discharge letters in the medical record.

Food intake and weight loss were similarly assessed as in the screening procedures. In addition, a single 24-h dietary recall was done following the USDA Automated Multiple Pass Method^24^, which measured the food intake when hospitalized. The collected data was inserted into “Kostholdsplanleggeren”, a dietary calculation tool based on the Norwegian Food Composition Table. The hospital meals were standardised in portion size and provided with nutritional value declaration, this was manually inserted into the analyses tool. For all other food items, including nutritional support, information was obtained from product packaging values and manufacturers’ websites and added to the calculation.

### Malnutrition diagnosis with the GLIM-criteria

The diagnosis of malnutrition was made retrospectively using the results from the study procedures in all patients, independent of the result of the nutritional risk screening. Calculations of BMI, self-reported weight loss during the last 3 months, and MAMC used as phenotypic criteria, and serum CRP and self-reported food intake the last week used as etiologic criteria (Table 1). Presence of inflammation was defined as CRP ≥ 5 mg/L^2^.

**Table 1:** Phenotypic and etiologic criteria applied in the nutritional assessment. Weight loss, BMI, MAMC, food intake and inflammation are suggested by GLIM for the diagnosis of malnutrition.

	Phenotypic criteria			Etiologic criteria	
	Weight loss within the past 3 months^1^	Body mass index (kg/m^2^)	Reduced muscle mass ^2^	Reduced food intake last week ^1^	Inflammation
Malnutrition^3^	> 5%	< 20 if < 70 years or < 22 if > 70 years	Any reduction in MAMC below 10th or 5th percentile	< 50% of normal	CRP ≥ 5 mg/dl
Moderate malnutrition	5–10%	< 20 if < 70 years or < 22 if > 70 years	Reduction in MAMC < 10th percentile	Not applicable	Not applicable
Severe malnutrition	> 10%	< 18,5 if < 70 years or < 20 if > 70 years	Reduction in MAMC < 5th percentile	Not applicable	Not applicable

GLIM, Global Leadership Initiative on Malnutrition; MAMC, mid-arm muscle circumference; CRP, c-reactive protein.

^1^Self-reported food intake < 50% of normal during the last week and self-reported weight loss > 5% within past 3 months from a study questionnaire.

^2^Age and sex specific cut-offs for MAMC were applied^22^.

^3^At least 1 phenotypic criterion and 1 etiologic criterion was required for the diagnosis of malnutrition.

### Statistical analysis

Continuous variables are presented as median with 25th and 75th percentile as the data were not normally distributed. The normality of the data was analyzed using the Shapiro–Wilk test. Categorical variables are presented as numbers and percentages. The Chi-square test was used to compare categorical variables, and the Mann–Whitney U-test was used for quantitative variables. A post hoc calculation of the required sample size at a power of 90% with a significance level of 5% when comparing two proportions was conducted, assuming a proportion of 44% patients identified by screening and 35% of patients identified by GLIM-criteria. This calculation revealed a required sample size of 289 patients.

All analyses were performed using IBM SPSS statistics version 26 (IBM corp., Armonk, NY, USA). A p-value of less than 0.05 was considered significant.

### Ethics

The study protocol was approved by the Regional Committee for Medical and Health Research Ethics –Western Norway (REC ref nr.: 2016/792) and conducted according to the ethical principles in the Declaration of Helsinki^25^. All participants received verbal and written information about the study, and signed informed consent was collected before participation.

## Results

**Figure 1:**
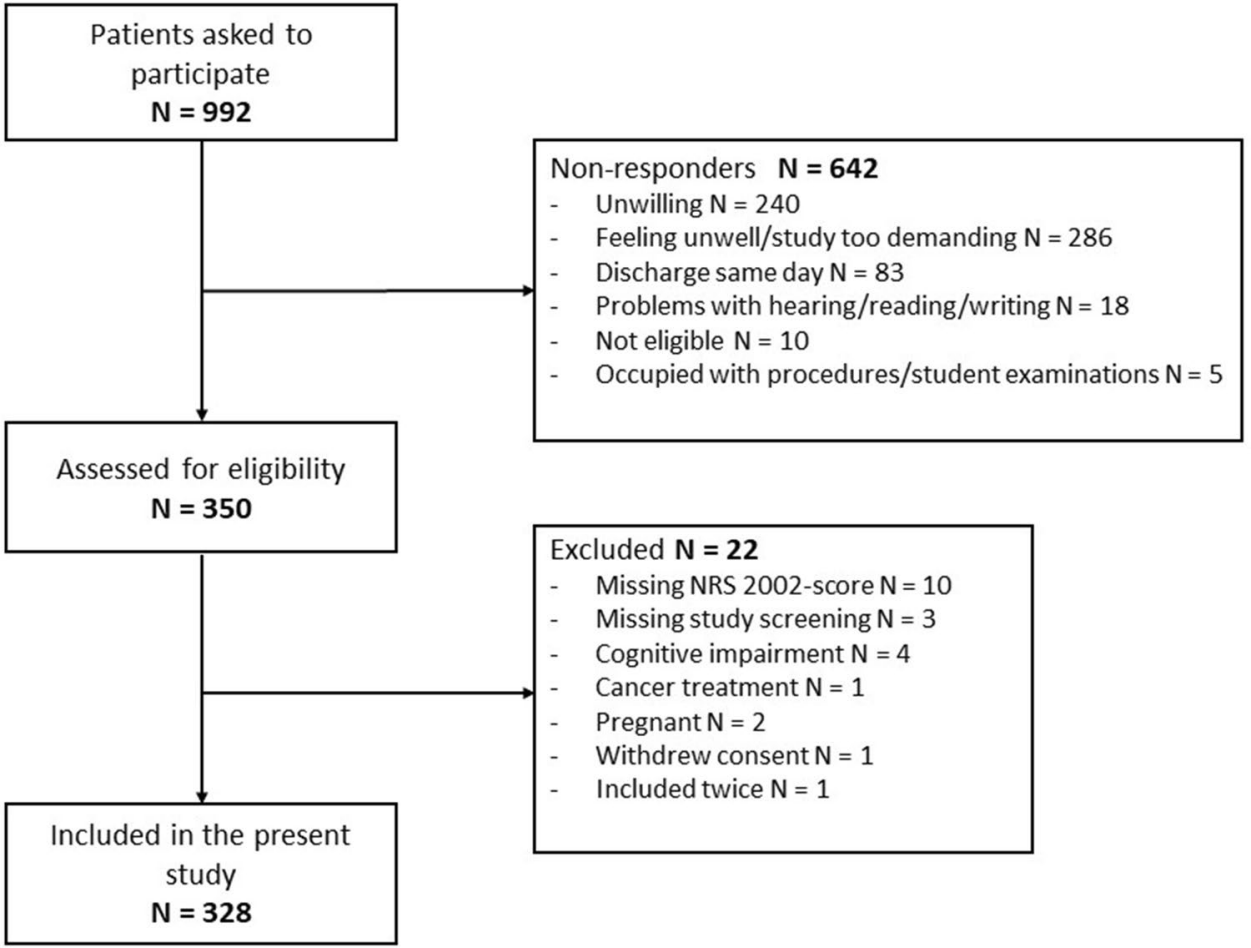
Flow chart of the recruitment and sample selection.

In total, 992 patients were asked to participate, and 35% (n = 350) provided informed consent for the study. Of these, 22 patients were excluded (Fig. 1), which resulted in a total of 328 patients included in the statistical analysis. The non-responders (n = 642) were at median 8 years older than the patients included in the study (results not shown). The patients included had a median age of 71 years, median bodyweight of 71 kg, and 47% were female (Table 2). Of the included patients, n = 52 or 16% were hospitalized for elective reasons.

**Table 2:** Characteristics of patients according to age groups. Continuous variables are presented as medians (25th, 75th percentile) and categorical variables are reported as counts (%).

		All patients	Age < 70 yr	Age ≥ 70 yr
		(n = 328)	(n = 152)	(n = 176)
Age^1^, yr		71 (60, 78)	59 (45, 65)	77 (74, 82)
Length of stay, days		7 (4, 11)	7 (3, 11)	7 (5, 11)
Number of diagnosis/Comorbidity		3 (2, 4)	3 (1, 4)	3 (2, 4)
Sex				
	Women	154 (47)	68 (45)	86 (49)
Departments				
	Heart Disease	111 (34)	44 (29)	67 (38)
	Thoracic Medicine	136 (41)	64 (42)	72 (41)
	Other^2^	81 (25)	44 (29)	37 (21)
Anthropometric and functional measurements				
	BMI, kg/m^2^	25 (22, 28)	24 (21, 28)	25 (22, 29)
	Bodyweight, kg			
	Women	64 (56, 72)	66 (59, 73)	62 (53, 73)
	Men	78 (67, 87)	80 (69, 89)	77 (66, 87)
	MAMC^3^, cm			
	Women	21 (19, 23)	22 (20, 24)	20 (19, 22)
	Men	24 (22, 27)	25 (23, 28)	23 (21, 26)
	HGS^3^, kg			
	Women	21 (18, 25)	24 (21, 30)	19 (16, 23)
	Men	35 (29, 42)	41 (34, 47)	30 (25, 37)
Blood samples				
	CRP, mg/L	15 (5, 50)	12 (3, 52)	17 (6, 49)
Energy intake, kcal/day				
	Women	1390 (1000, 1827)	1555 (907, 1995)	1335 (1021, 1579)
	Men	1801 (1306, 2170)	1915 (1371, 2347)	1665 (1174, 2090)
Protein intake, g/day				
	Women	53 (35, 71)	62 (37, 81)	49 (34, 65)
	Men	65 (44, 88)	72 (51, 96)	60 (38, 77)
Results from study questionnaire^4^				
	Low food intake last week^3^	61 (20)	31 (22)	30 (18)
	Weight loss last 3 months^3^	101 (32)	51 (35)	50 (30)

BMI, Body mass index; CRP, C-reactive protein; HGS, handgrip strength; GLIM, Global Leadership Initiative on Malnutrition^1^Age at inclusion.

^2^Other departments: Gastroenterology, Endocrinology, Dermatology and Rheumatology, and the Orthopedic Clinic.

^3^Missing values: n = 305 patients with information on MAMC; n = 291 patients with information on HGS; n = 304 patients with information on low food intake the last week; n = 312 patients with information on weight loss the last 3 months.

^4^Self-reported food intake < 50% of normal during the last week and self-reported weight loss > 5% within past 3 months from a study questionnaire.

### Energy and protein intake and nutritional support

Energy and protein intake were related to age and to risk of malnutrition, both in men and women, with lower intakes in older patients and in those at risk of malnutrition or with malnutrition. The percentage of patients receiving oral nutritional supplements was 9%, and 3% of the patients received a nutritional treatment plan. Among the patients at risk of malnutrition, 18% received oral nutritional supplements (ONS), which contributed 22% of the total energy intake of these patients. Figures for malnutrition were 17% of patients with ONS, which supplied 22% of total energy intake (Supplemental Tables 1 and 2).

### Nutritional risk screening

The screening identified 143 patients at nutritional risk (71 women and 72 men). Patients at risk of malnutrition were older (median age 74 vs. 68 years), had lower bodyweight (median 56 vs. 70 kg in women, and 68 vs. 82 kg in men), and lower BMI (median 22 vs. 27 kg/m^2^) compared to patients not at risk. Levels of CRP were significantly higher in patients at risk than in patients not at risk and MAMC and HGS were significantly lower both in women and men in the at risk group compared to patients not at risk. Energy intake did not significantly differ; however, protein intake was significantly lower in patients at risk of malnutrition compared to patients not at risk (Table 3). Results were not substantially changed when elective patients were excluded from the analysis (Supplemental Table 3).

**Table 3:** Patient characteristics according to nutritional status assessed by the GLIM-criteria and according to the results from the study nutritional risk screening.

		Nutritional assessment (GLIM)^1^			Nutritional risk screening (NRS2002)		
		No malnutrition	Malnutrition	*P*-value^2^	Not at risk	At risk	*P*-value^2^
		(n = 212)	(n = 114)		(n = 185)	(n = 143)	
General characteristics							
	Women, n (%)	111 (52)	42 (37)	0.007^3^	83 (45)	71 (50)	0.389^3^
	Age^2^, ≥ 70 years, n (%)	111 (52)	64 (56)	0.514^3^	83 (45)	93 (65)	< 0.001^3^
Age, yr		70 (58, 78)	72 (64, 78)	0.307	68 (57, 75)	74 (64, 80)	< 0.001
Anthropometric and functional measurements							
BMI, kg/m^2^		27 (24, 30)	21 (19, 24)	0.000	27 (23, 30)	22 (19, 25)	< 0.001
MAMC^4^, cm							
	Women	22 (20, 24)	19 (18, 21)	0.000	22 (20, 25)	20 (19, 21)	< 0.001
	Men	26 (24, 27)	22 (20, 23)	0.000	25 (23, 27)	23 (21, 25)	< 0.001
HGS^4^, kg							
	Women	22 (19, 27)	19 (16, 23)	0.004	24 (20, 29)	19 (16, 23)	< 0.001
	Men	38 (30, 45)	32 (26, 39)	0.005	38 (30, 44)	34 (26, 40)	0.018
Blood samples							
CRP, mg/L		11 (3, 43)	28 (11, 79)	0.000	12 (3, 39)	23 (5, 69)	0.001
Energy intake, kcal/day							
	Women	1478 (1041, 1891)	1245 (913, 1648)	0.221	1478 (1039, 1893)	1300 (897, 1648)	0.086
	Men	1876 (1399, 2233)	1639 (1137, 2094)	0.090	1849 (1405, 2174)	1640 (1064, 2163)	0.112
Protein intake, g/day							
	Women	59 (41, 77)	47 (32, 59)	0.042	59 (42, 80)	48 (31, 64)	0.014
	Men	66 (45, 90)	60 (43, 84)	0.331	69 (50, 90)	58 (33, 84)	0.006
Results from study questionnaire^5^							
Low food intake last week, n (%)		29 (14)	30 (26)	0.012^3^	7 (0.5)	54 (38)	< 0.001^3^
Weight loss last 3 months, n (%)		37 (17)	64 (56)	0.000^3^	25 (14)	76 (53)	< 0.001^3^

Continuous variables are presented as medians (25th, 75th percentile) and categorical variables are reported as counts (%).

BMI, Body mass index; CRP, C-reactive protein; HGS, handgrip strength; GLIM, Global Leadership Initiative on Malnutrition; MAMC, Mid-arm muscle circumference.

^1^Two patients could not be assessed due to missing information.

^2^P-value is calculated using Mann–Whitney U-test for nonparametric independent samples, significance level: *p* < 0.05.

^3^*P*-value is calculated using Pearson’s chi-square test, significance level: *p* < 0.05.

^4^Missing values: n = 305 patients with information on MAMC; n = 291 patients with information on HGS; n = 304 patients with information on low food intake the last week; n = 312 patients with information on weight loss the last 3 months.

^5^Self-reported food intake < 50% of normal during the last week and self-reported weight loss > 5% within past 3 months from a study questionnaire.

### Applying GLIM criteria for malnutrition

An evaluation of the diagnosis of malnutrition was done in all patients regardless of the screening result by applying the second step of GLIM criteria, and it led to a diagnosis of malnutrition in 114 patients (42 women and 72 men). Patients with malnutrition had lower body weight and BMI, lower MAMC and HGS, and higher CRP compared to patients who were not malnourished, while age and energy intake in the 24-h dietary recall did not differ (Table 3). In total, 41 patients were moderately malnourished and 73 patients were severely malnourished (data not shown).

### Comparison of screening for risk of malnutrition and diagnosis of malnutrition

Of the 143 patients who were at risk of malnutrition by screening, 77 (54%) were also diagnosed as malnourished by the GLIM criteria. Patients who were at risk for malnutrition but did not get a diagnosis (n = 65) included more women and were older, had higher BMI and body weight, had higher muscle mass and function, lower inflammation, and lower prevalence of weight loss than those at risk of malnutrition and a diagnosis of malnutrition (n = 77) (Table 4).

**Table 4:** Patients’ characteristics according to results of nutritional assessment (GLIM) and nutritional risk screening (NRS2002). Malnutrition was diagnosed by nutritional assessment in 114 patients, of these, 77 were also identified by NRS2002. Malnutrition was not confirmed in 65 patients at risk of malnutrition. For comparison, also those not at risk and not diagnosed with malnutrition are presented.

		Malnourished patients		Patients at risk of malnutrition	
		Malnourished but not at risk	Malnourished and at risk	At risk but not malnourished	Not at risk and not malnourished
		(n = 37)	(n = 77)	(n = 65)	(n = 147)
*Age*^*1*^*, yr*		69 (58, 74)	72 (65, 79)	74 (63, 82)	67 (57, 76)
*Sex*					
	Women	11 (30)	31 (40)	40 (62)	71 (48)
**Anthropometric and functional measurements**					
BMI, kg/m^2^		23 (21, 26)	20 (18, 23)	24 (22, 27)	28 (25, 30)
Bodyweight, kg					
	Women	62 (57, 66)	52 (48, 56)	62 (53, 70)	72 (61, 82)
	Men	74 (68, 85)	64 (58, 75)	80 (67, 89)	84 (78, 96)
MAMC^2^, cm					
	Women	19 (19, 22)	18 (17, 20)	20 (20, 23)	23 (21, 25)
	Men	22 (20, 23)	21 (20, 24)	24 (23, 27)	26 (24, 28)
HGS^2^, kg					
	Women	23 (19, 27)	18 (14, 21)	20 (16, 23)	24 (20, 29)
	Men	32 (28, 41)	33 (25, 38)	35 (28, 42)	38 (31, 46)
**Blood samples**					
	CRP, mg/L	23 (8, 53)	31 (15, 99)	12 (3, 49)	11 (3, 36)
Energy intake, kcal/day					
	Women	1196 (819, 1596)	1301 (951, 1658)	1325 (872, 1680)	1544 (1154, 1922)
	Men	1588 (1165, 2068)	1641 (1029, 2171)	1619 (1083, 2139)	1918 (1437, 2317)
Protein intake, g/day					
	Women	43 (32, 52)	49 (33, 61)	48 (34, 66)	64 (45, 80)
	Men	69 (45, 85)	58 (38, 86)	50 (29, 81)	69 (52, 90)
Results from study questionnaire^*3*^					
Low food intake last week^2^		2 (5)	29 (38)	25 (38)	5 (3)
Weight loss last 3 months^2^		10 (27)	54 (70)	22 (34)	15 (10)

Continuous variables are presented as medians (25th, 75th percentile) and categorical variables are reported as counts (%).

BMI, Body mass index; CRP, C-reactive protein; GLIM, Global Leadership Initiative on Malnutrition; HGS, handgrip strength; NRS2002, Nutritional Risk Screening 2002; MAMC, Mid-arm muscle circumference.

^1^Age at inclusion.

^2^Missing values: n = 305 patients with information on MAMC; n = 304 patients with information on low food intake the last week; n = 312 patients with information on weight loss the last 3 months.

^3^Self-reported food intake < 50% of normal during the last week and self-reported weight loss > 5% within past 3 months from a study questionnaire.

On the other hand, applying the GLIM diagnostic assessment in all patients, lead to the identification of 37 of 185 (20%) patients as malnourished who have not been identified by screening. Thus, 77 (68%) of those with a diagnosis of malnutrition were identified by nutritional risk screening. Patients who are malnourished but have not been identified by NRS2002 (n = 37) were younger, had higher BMI and body weight, and lower prevalence of weight loss during the last 3 months than the malnourished patients identified by NRS2002 (Table 4).

## Discussion

It was the aim of this study to investigate nutritional status in a population of hospitalized patients in a tertiary hospital. Trained study personnel performed the nutritional screening using NRS2002, followed by the GLIM diagnostic assessment in all patients independent of the result of screening procedure. This led to a diagnosis of malnutrition in 114 (35%) patients, and of these, 77 have also been identified by NRS2002 as at risk of malnutrition, which would give a final prevalence of 24% of included patients who were malnourished if the GLIM diagnostic criteria have only been applied to patients at risk. This observed prevalence is in line with other studies reporting a range of 19–80% when applying the GLIM-criteria in a hospital setting^26–31^. The wide range of prevalence can also be explained by the lack of standardization regarding nutritional screening tools and methods applied to assess reduced muscle mass and inflammation^32,33^. Indeed, we used MAMC for the measurement of low muscle mass due to the lack of bioelectrical impedance measurements in about 30% of our patients. MAMC has been shown to be associated with mortality^34^ but has not been widely used in hospital studies of malnutrition.

A diagnosis of malnutrition was confirmed in 54% of the patients at risk of malnutrition. There are few studies that have investigated this, and thus it is difficult to interpret this proportion. NRS2002 recommends to re-screen patients not at risk after 1 week, as nutritional status in the hospital can change rapidly and malnutrition can develop. However, the median length of stay in our study was 7 days, thus, re-screening would not be applicable in half of the patients. Patients at risk with no subsequent diagnosis of malnutrition were older, had higher BMI and body weight, and lower weight loss history than patients at risk who received a diagnosis of malnutrition. More importantly, they had higher MAMC and lower CRP levels, which became only evident applying the diagnostic criteria.

On the other hand, we observed that also patients were diagnosed as malnourished applying the second step of GLIM who had not been identified by nutritional risk screening. If the diagnosis of malnutrition would have followed both steps of GLIM, these patients thus would not have been identified. This result can be due to that either the screening process is not sensitive enough or that the diagnosis of malnutrition is using inappropriate criteria. Patients not at risk but with a diagnosis of malnutrition were younger and had higher BMI and body weight and lower prevalence of weight loss history than those at risk with a diagnosis, and MAMC was similar. Thus, low muscle mass was relevant for the diagnosis, which is not assessed in the screening procedures. CRP levels in patients not at risk with a diagnosis of malnutrition were also lower than in patients at risk with a diagnosis of malnutrition, suggesting that inflammation measured by CRP also was relevant for the diagnosis. Although screening for risk of malnutrition and diagnosing malnutrition are two different procedures, both NRS2002, and the GLIM-criteria use some of the same measurements, and regarding these measures, we observed, as expected, rather similar results. The main novelty of the GLIM-criteria is the inclusion of assessment of muscle mass, which we found to be relevant.

Concerning muscle mass and function, they are both strongly linked with sarcopenia, cachexia, and frailty and have been shown to be related to mortality^34–37^. GLIM decided to use muscle mass as a phenotypic criteria and HGS as a supportive measure. Thus, we also used MAMC and not HGS for the diagnosis of malnutrition.

From our results, it is suggested that the measurement of muscle mass and function can provide important information on the nutritional status of the patient.

### Strengths and limitations

Our study has several strengths and limitations. First, the study screening was performed by trained personnel, which may lead to differences compared to the routine clinical situation. All patients underwent the GLIM diagnostic assessment regardless of the screening result. We used standardized questionnaires and instruments for the data collection and measurements. However, many methods and cut-offs which are used in the GLIM process are still under discussion, and this may limit the generalizability of our data. It is also a limitation that we were missing BIA measurements in about 30% of the patients, which is one of the technology-based measurements primarily recommended by GLIM, leaving the more clinical approach of measuring MAMC as our best available measure of muscle mass. The results on energy and protein intake in our study are from a single 24-h dietary recall, which limits their interpretation of nutritional intake and nutritional status.

It can be discussed whether the selection of patients is a strength or a limitation. Our study excluded patients with acute diagnosis or treatment of cancer, patients from any intensive care units, and those with transmissible infections. However, we cannot exclude that some patients had an earlier diagnosis or treatment of cancer. Cancer, ICU treatment and transmissible infections are associated with very high prevalence and incidence of malnutrition^9,38,39^, and this may have influenced the results. Our study was not designed to assess the prevalence of overall nutritional risk or malnutrition at the included wards, and thus we cannot provide prevalence figures.

## Conclusions

Malnutrition was diagnosed in 54% of patients identified at risk by NRS2002 in a tertiary hospital. In addition, malnutrition was also diagnosed in 20% of the patients not at risk by NRS2002. A comparison of nutritional screening results and diagnosis of malnutrition is thus warranted in further studies with larger numbers of patients and different patients’ selection criteria to further elucidate the importance of inflammation and reduced muscle mass, which is the main difference between nutritional risk screening and GLIM diagnostic assessment.

## Supplementary Information

Supplementary Information 1.

Supplementary Information 2.

## Data Availability

Additional data are available from the corresponding author on reasonable request.
