# Supplementary material for: Comparison of nutritional risk screening with NRS2002 and the GLIM diagnostic criteria for malnutrition in hospitalized patients
Source: Sci Rep. 2022 Nov 17;12:19743. doi: 10.1038/s41598-022-23878-3 (PMC9672100; doi:10.1038/s41598-022-23878-3)
Supplement: Supplementary file 1 — Supplementary Information 1. [file 41598_2022_23878_MOESM1_ESM.docx]

Supplemental Table 1: Overview on nutritional support to included patients according to nutritional risk status.

|  | All patients | NRS 2002 at risk | NRS 2002 not at risk |
| --- | --- | --- | --- |
|  | (n = 328) | (n = 143) | (n = 185) |
| Mean energy intake, kcal (± SD) | 1588 (664) | 1487 (674) | 1663 (648) |
| Number of patients recieving oral nutritional supplements (%) | 28 (9) | 26 (18) | 2 (1) |
| Median E% oral nutritional supplements (ONS) contributed to total energy intake in the patients receiving ONS | 22% | 22% | 23% |
| Number of patients receiving a nutritional treatment plan (%) | 11 (3) | 11 (8) | 0 (0) |
| Number of patients being referred to a dietician (%) | 10 (3) | 10 (7) | 0 (0) |
| Number of patients recieving a ICD-10 diagnosis of malnutrition (%) | 14 (7) | 14 (10) | 0 (0) |

Supplemental Table 2: Overview on nutritional support to included patients according to nutritional status according to the GLIM-criteria.

|  | GLIM malnutrition | GLIM no malnutrition |
| --- | --- | --- |
|  | (n = 114) | (n = 212) |
| Mean energy intake, kcal (± SD) | 1549 (628) | 1630 (663) |
| Number of patients recieving oral nutritional supplements (%) | 19 (17) | 9 (4) |
| Median E% oral nutritional supplements (ONS) contributed to the total energy intake in the patients receiving ONS | 22% | 24% |
| Number of patients receiving a nutritional treatment plan (%) | 11 (10) | 0 (0) |
| Number of patients being referred to a dietician (%) | 8 (7) | 2 (1) |
| Number of patients receiving a ICD-10 diagnosis of malnutrition (%) | 10 (9) | 3 (1) |
