# Supplementary material for: Comparison of nutritional risk screening with NRS2002 and the GLIM diagnostic criteria for malnutrition in hospitalized patients
Source: Sci Rep. 2022 Nov 17;12:19743. doi: 10.1038/s41598-022-23878-3 (PMC9672100; doi:10.1038/s41598-022-23878-3)
Supplement: Supplementary file 2 — Supplementary Information 2. [file 41598_2022_23878_MOESM2_ESM.docx]

Supplemental Table 3: Screening for nutritional risk and diagnosis of malnutrition limited to the patients with acute admission (n=276)

|  |  | **Nutritional assessment (GLIM)^1^** | |  | **Nutritional risk screening (NRS2002)** | |  |
| --- | --- | --- | --- | --- | --- | --- | --- |
|  |  | **No malnutrition** | **Malnutrition** | **P-value^2^** | **Not at risk** | **At risk** | **P-value^2^** |
|  |  | **(n = 175)** | **(n = 99)** |  | **(n = 147)** | **(n = 129)** |  |
| General characteristics | |  |  |  |  |  |  |
|  | Women, n (%) | 86 (49) | 34 (34) | 0.018^3^ | 61 (41) | 61 (47) | 0.336^3^ |
|  | Age^2^, ≥ 70 years, n (%) | 97 (55) | 58 (59) | 0.514^3^ | 72 (49) | 85 (66) | 0.005^3^ |
|  |  |  |  |  |  |  |  |
| Age, yr | | 71 (60, 78) | 72 (64, 79) | 0.411 | 69 (59, 76) | 74 (64, 81) | <0.001 |
| Anthropometric and functional measurements | |  |  |  |  |  |  |
| BMI, kg/m^2^ | | 27 (24, 30) | 21 (19, 24) | <0.001 | 27 (23, 30) | 22 (19, 25) | <0.001 |
| MAMC^4^, cm | |  |  |  |  |  |  |
|  | *Women* | 22 (20, 24) | 19 (18, 21) | <0.001 | 22 (20, 24) | 20 (19, 22) | <0.001 |
|  | *Men* | 25 (24, 27) | 21 (20, 23) | <0.001 | 24 (23, 27) | 23 (21, 25) | 0.001 |
| HGS^4^, kg | |  |  |  |  |  |  |
|  | *Women* | 23 (19, 27) | 18 (14, 22) | 0.001 | 23 (19, 29) | 19 (16, 23) | <0.001 |
|  | *Men* | 38 (30, 43) | 32 (25, 39) | 0.009 | 37 (30, 43) | 33 (27, 40) | 0.047 |
| Blood samples | |  |  |  |  |  |  |
| CRP, mg/L | | 13 (3, 47) | 29 (11, 94) | <0.001 | 13 (6, 46) | 23 (6, 75) | 0.014 |
| Energy intake, kcal/day | |  |  |  |  |  |  |
|  | *Women* | 1443 (1021, 1806) | 1245 (897, 1596) | 0.362 | 1417 (1062, 1830) | 1301 (881, 1596) | 0.210 |
|  | *Men* | 1876 (1403, 2272) | 1636 (1144, 2084) | 0.062 | 1865 (1428, 2220) | 1619 (1059, 2116) | 0.023 |
| Protein intake, g/day | |  |  |  |  |  |  |
|  | *Women* | 53 (35, 71) | 48 (37, 61) | 0.244 | 53 (42, 73) | 49 (33, 64) | 0.133 |
|  | *Men* | 67 (42, 90) | 59 (43, 84) | 0.470 | 70 (52, 90) | 56 (33, 80) | 0.002 |
| Results from study questionnaire^5^ | |  |  |  |  |  |  |
| Low food intake last week, n (%) | | 29 (17) | 27 (27) | 0.068^3^ | 5 (0.5) | 51 (40) | <0.001^3^ |
| Weight loss last 3 months, n (%) | | 30 (17) | 53 (54) | <0.001^3^ | 16 (11) | 67 (52) | <0.001^3^ |
|  |  |  |  |  |  |  |  |
| ^1^Two patients could not be assessed due to missing information. | | | |  |  |  |  |
| ^2^P-value is calculated using Mann-Whitney U-test for nonparametric independent samples, significance level: p < 0.05 | | | | | | |  |
| ^3^P-value is calculated using Pearson’s chi-square test, significance level: p < 0.05 | | | | |  |  |  |
| ^4^Missing values: n = 305 patients with information on MAMC; n = 291 patients with information on HGS; n = 304 patients with information on low food intake the last week; n = 312 patients with information on weight loss the last 3 months. | | | | | | | |
| ^5^Self-reported food intake <50% of normal during the last week and self-reported weight loss >5% within past 3 months from a study questionnaire. | | | | | | | |
| *Abbreviations: BMI; Body mass index, CRP; C-reactive protein, HGS; handgrip strength, GLIM; Global Leadership Initiative of Malnutrition, MAMC; Mid-arm muscle circumference.* | | | | | | | |
